# Quercetin Inhibits Orthodontic Tooth Movement‐Associated Periodontitis by Regulating Macrophage Aerobic Glycolysis Through the IL‐17/Hippo/YAP Axis

**DOI:** 10.1002/cre2.70383

**Published:** 2026-05-26

**Authors:** Fen Yao, Zhiping Song, Wuchao Wu, Bingqian Liu, Rongqi Xia, Yao Zhou, Wenxia Wang, Yujie Chen, Zhengyu Liao

**Affiliations:** ^1^ Jiangxi Provincial Key Laboratory of Oral Diseases, Jiangxi Provincial Clinical Research Center for Oral Diseases The Affiliated Stomatological Hospital, Jiangxi MedicalCollege, Nanchang University Nanchang Jiangxi China; ^2^ Department of Anesthesia, The First Affiliated Hospital, Jiangxi Medical College Nanchang University Nanchang Jiangxi China

**Keywords:** Hippo/YAP signaling, IL‐17, OTM, quercetin

## Abstract

**Objectives:**

Orthodontic tooth movement (OTM) is frequently accompanied by inflammatory responses, including periodontitis, which may compromise treatment outcomes. This study investigated the protective role of quercetin, a natural flavonoid, in OTM‐associated periodontitis and its underlying mechanism involving macrophage metabolic reprogramming via the IL‐17/Hippo/YAP axis.

**Material and Methods:**

An in vivo OTM model was established using YAP‐wild‐type and YAP‐knockout Sprague‐Dawley rats to assess the effects of quercetin on periodontal inflammation and alveolar bone resorption. In vitro studies were conducted using the RAW264.7 macrophage cell line treated with quercetin (0.1–10 μM). Key methodologies included micro‐CT analysis for alveolar bone loss, histopathological evaluation (H&E and TRAP staining), qPCR and western blot analysis for gene and protein expression, ELISA for cytokine quantification, flow cytometry for apoptosis and cell cycle analysis, and CCK‐8 assays for cell viability. The role of YAP was further validated using specific inhibitors and genetic knockout models.

**Results:**

Quercetin administration significantly attenuated periodontal inflammation and alveolar bone loss in the OTM model. In RAW264.7 macrophages, quercetin suppressed IL‐17‐induced activation of the Hippo/YAP pathway, inhibited YAP nuclear translocation, and downregulated the expression of key glycolytic enzymes (HK2, LDHA, and PKM2). Consequently, quercetin reduced lactate production and aerobic glycolysis. These effects were reversed upon YAP overexpression, confirming the pivotal role of the IL‐17/Hippo/YAP axis in quercetin‐mediated metabolic modulation.

**Conclusions:**

Quercetin alleviates OTM‐associated periodontitis by inhibiting the IL‐17/Hippo/YAP signaling pathway, thereby suppressing macrophage aerobic glycolysis. These findings highlight quercetin as a potential therapeutic adjunct in orthodontics to mitigate inflammatory complications.

## 1 Introduction

Orthodontic tooth movement (OTM) is a dynamic biological process involving mechanical force‐induced bone remodeling and periodontal tissue adaptation (Aline et al. 2025). While essential for achieving functional and aesthetic dental alignment, OTM is frequently accompanied by localized inflammatory responses, including periodontitis, which may exacerbate tissue damage, delay treatment progress, and compromise long‐term stability (Behzad et al. 2025). Periodontitis associated with orthodontic forces is characterized by alveolar bone resorption, gingival inflammation, and immune cell infiltration, particularly macrophages—a central player in orchestrating both inflammatory and reparative phases of periodontal health (Rawand and Omar 2024). Emerging evidence highlights that macrophage polarization and metabolic reprogramming are pivotal in driving the balance between tissue destruction and repair (Xufeng et al. 2025). Among metabolic pathways, aerobic glycolysis (the “Warburg effect”) has gained attention as a hallmark of pro‐inflammatory macrophage activation, fueling inflammatory cytokine production and osteoclastogenesis (G. Qing et al. 2025). However, the molecular mechanisms linking mechanical stress, immune‐metabolic dysregulation, and periodontal inflammation during OTM remain poorly understood, underscoring the need for targeted therapeutic strategies to mitigate these complications.

The interleukin‐17 (IL‐17) signaling pathway has emerged as a critical mediator of periodontal inflammation. IL‐17, primarily secreted by Th17 cells and innate lymphoid cells, amplifies inflammatory cascades by promoting neutrophil recruitment, osteoclast differentiation, and pro‐inflammatory cytokine release in periodontal tissues (Xingling et al. 2025). Recent studies further implicate IL‐17 in metabolic reprogramming of macrophages, particularly through crosstalk with the Hippo/Yes‐associated protein (YAP) pathway—a conserved signaling network regulating cell proliferation, apoptosis, and metabolism (Shumei et al. 2025). The Hippo/YAP axis, governed by kinases MST1/2 and LATS1/2, controls YAP nuclear translocation, where it partners with transcription factors to modulate genes involved in glycolysis, inflammation, and tissue remodeling (Jiani et al. 2024). Intriguingly, mechanical forces, such as those applied during OTM, can activate YAP, suggesting a potential link between orthodontic stress, IL‐17‐driven inflammation, and Hippo/YAP‐mediated metabolic shifts in macrophages. Despite these advances, the role of the IL‐17/Hippo/YAP axis in regulating macrophage glycolysis and its contribution to OTM‐associated periodontitis remains unexplored.

Quercetin, a bioactive flavonoid abundant in fruits and vegetables, has garnered interest for its anti‐inflammatory, antioxidant, and metabolic‐modulating properties (Xinxin et al. 2025). Preclinical studies demonstrate its efficacy in attenuating periodontitis by suppressing NF‐κB and MAPK pathways, reducing oxidative stress, and inhibiting osteoclastogenesis (Xinxin et al. 2025). Notably, quercetin has been shown to interfere with glycolytic metabolism in cancer cells and immune cells, suggesting its potential to target metabolic reprogramming in inflammatory macrophages (L. Qing et al. 2025; Xue et al. 2024). However, whether quercetin exerts protective effects against OTM‐associated periodontitis by modulating macrophage glycolysis via the IL‐17/Hippo/YAP axis remains unknown. Addressing this gap could provide novel insights into the metabolic regulation of periodontal inflammation and identify quercetin as a therapeutic adjunct in orthodontics. By bridging the gap between mechanical stress, immune cell metabolism, and periodontal pathology, this study advances our understanding of the molecular underpinnings of OTM complications. It also highlights the therapeutic potential of targeting metabolic pathways to control inflammation, offering a paradigm shift in managing orthodontic treatment‐related side effects.

## 2 Materials and Methods

### 2.1 Animal Models

A total of sixty 6‐week‐old healthy male Sprague‐Dawley (SD) rats, comprising 30 YAP‐wild‐type (YAP WT) and 30 YAP‐knockout (YAP KO) rats with body weights ranging from 180 to 250 g, were procured from The First Affiliated Hospital of Nanchang University. All experimental procedures were conducted in compliance with international guidelines for animal research. Following a 3‐day acclimatization period under standard circadian conditions in a group‐housing environment, rats of each genotype were randomly assigned to five experimental groups (*n* = 6 per group).

### 2.2 Cell Culture

The RAW264.7 macrophage cell line was cultured and maintained in the logarithmic growth phase. Cells were categorized into a control group and an experimental group. Experimental groups were treated with quercetin at specified concentrations (0.1, 1, and 10 μM), while the control group received an equivalent volume of standard culture medium. All cells were maintained in Dulbecco's Modified Eagle Medium (DMEM) supplemented with 10% fetal bovine serum, within a humidified incubator at 37°C with 5% CO_2_.

### 2.3 Quantitative RT‐PCR Assay

Total RNA was extracted and reverse‐transcribed into cDNA following established protocols (L. Qing et al. 2025). Quantitative PCR (qPCR) was performed using a LightCycler system (Roche, USA) with a SYBR RT‐PCR kit (Takara, Japan). Relative gene expression levels were calculated via the 2^−ΔΔCt^ method, normalized to the endogenous control β‐actin, and compared to the control group.

### 2.4 Hematoxylin and Eosin Staining

Tissue samples were fixed in 95% ethanol for 20 min and subsequently washed twice with phosphate‐buffered saline (PBS) for 1 min per wash. Samples were then stained with hematoxylin for 2–3 min and rinsed under deionized water. Nuclear staining was differentiated using a 1% hydrochloric acid–alcohol solution for several seconds, followed by a wash with deionized water. Counterstaining was performed using eosin for 1 min, after which samples were rinsed with deionized water. Following air‐drying, tissue sections were mounted with neutral gum.

### 2.5 Tartrate‐Resistant Acid Phosphatase Staining

For Tissue Sections: Mandible samples were decalcified and embedded in paraffin. Sections were baked, deparaffinized, and rehydrated prior to staining with hematoxylin for 5 min. After rinsing with deionized water, sections were differentiated in 1% hydrochloric acid–alcohol, blued, and counterstained with eosin for 5 min. Following dehydration, sections were incubated with tartrate‐resistant acid phosphatase (TRAP) incubation solution (G1492, Solarbio) in a humidified chamber at 37°C for 60 min. Sections were then washed three times with PBS (3 min per wash), stained with methyl green for 3 min, washed again with PBS, and mounted for observation under a microscope (SC2000C, Nikon).

For Cultured Cells: Cell monolayers were washed three times with PBS (3 min per wash) and fixed with 4% paraformaldehyde for 15 min. After washing with PBS, cells were incubated with TRAP incubation solution at 37°C for 60 min. Following incubation, cells were rinsed three times with PBS, stained with methyl green for 3 min, washed, and observed under a microscope (BX43, OLYMPUS).

### 2.6 Flow Cytometry (Cell Cycle and Apoptosis)

Apoptosis Assay: Approximately 1 × 10^6^ cells were collected, centrifuged at 1500 rpm for 3 min, and washed twice with PBS. Cells were resuspended in 300 μL of pre‐cooled 1× Binding Buffer, followed by the addition of 5 μL Annexin V‐FITC and 10 μL propidium iodide (PI) (AP101‐100‐kit, MULTI SCIENCES). After gentle mixing, samples were incubated at room temperature in the dark for 10 min. Subsequently, 200 μL of pre‐cooled 1× Binding Buffer was added to each tube, and analysis was performed using a flow cytometer (NovoCyte 2060R, Agilent).

Cell Cycle Analysis: Approximately 1 × 10^6^ cells were harvested, centrifuged at 1500 rpm for 3 min, and washed with PBS. The cell pellet was resuspended in 1 mL of DNA staining solution containing 10 μL permeabilization solution, vortexed for 10 seconds, and incubated at room temperature in the dark for 30 min prior to analysis by flow cytometry.

### 2.7 Assessment of Alveolar Bone Loss

Maxillary samples were scanned using a Skyscan1276 Micro‐CT system (Bruker) operated via the associated Scanner software. Scans were performed at 70 kV and 200 μA with an isotropic resolution of 10.2 μm. Three‐dimensional reconstruction and analysis were conducted using CT‐AN software to quantify the alveolar bone loss (ABL) rate at the distal root of the left maxillary first molar.

### 2.8 Enzyme‐Linked Immunosorbent Assay

Purified antibody was diluted to a concentration of 1–10 μg/mL in carbonate coating buffer (0.05 M, pH 9.6). Each well of a polystyrene microplate was coated with 0.1 mL of the antibody solution and incubated overnight at 4°C. Following incubation, plates were washed three times with washing buffer. Subsequently, 0.1 mL of serially diluted sample or standard was added to duplicate wells and incubated at 37°C for 1 hour. After washing, 0.1 mL of freshly prepared TMB substrate solution was added to each well and incubated at 37°C for 10–30 min. The reaction was terminated by adding 0.05 mL of 2 M sulfuric acid. Absorbance was measured at 450 nm using a microplate reader, and sample concentrations were determined from the standard curve.

### 2.9 Western Blot Analysis

Protein samples were extracted and quantified using a BCA Protein Assay Kit (Abcam, USA). Equal amounts of protein were separated by SDS‐polyacrylamide gel electrophoresis and transferred onto a PVDF membrane (Thermo Fisher, USA). The membrane was blocked with 5% non‐fat milk and then incubated overnight at 4°C with the appropriate primary antibody (Abcam, USA; 1:1000 dilution). After washing three times with Tris‐buffered saline containing Tween 20 (TBST), the membrane was incubated with a horseradish peroxidase‐conjugated secondary antibody (Abcam, USA; 1:5000 dilution) at room temperature for 1 hour. Following additional washes with TBST, protein bands were visualized using an enhanced chemiluminescence (ECL) kit (Thermo Fisher, USA) and a digital imaging system.

### 2.10 Cell Counting Kit‐8 Assay

Cell suspensions were seeded into 96‐well plates at a density of 1 × 10^4^ cells per well and pre‐incubated for 24 hours. Subsequently, 10 μL of Cell Counting Kit‐8 (CCK‐8) reagent (Abcam, USA) was added to each well. After incubation at 37°C for 1 hour, absorbance was measured at 450 nm using a microplate reader.

### 2.11 Statistical Analysis

All data are presented as mean ± standard error of the mean (SEM). Statistical analysis was performed using GraphPad Prism 8.1 software. Differences among groups were evaluated by one‐way analysis of variance (ANOVA) followed by Tukey's post hoc test. A *p*‐value of less than 0.05 was considered statistically significant. All experiments were performed in triplicate.

## 3 Results

### 3.1 Quercetin Ameliorates Orthodontic Force‐Induced Periodontal Inflammation and Alveolar Bone Resorption In Vivo

**Figure 1:**
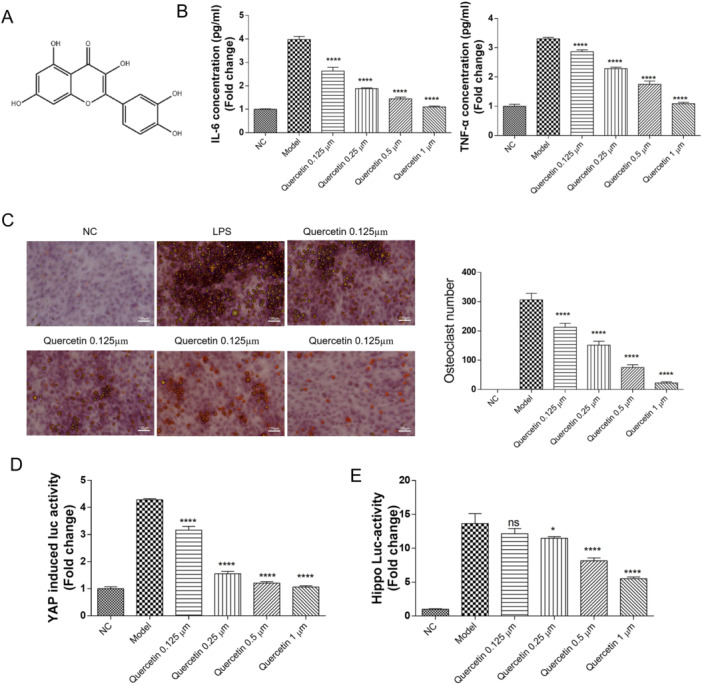
Quercetin inhibits LPS‐induced osteoclastogenesis. (A) The chemical structure of artesunate. (B) Quercetin inhibits LPS‐induced IL‐6 and TNF‐α expression. (C) Quercetin inhibits LPS‐induced osteoclast formation as assessed by a TRAP assay in preosteoclastic RAW264.7 cells. (D, E) Quercetin inhibits LPS‐induced Hippo/YAP activity. Data are mean ± s.e.m., *n* = 3, *p < 0.05, ***p < 0.001, ****p < 0.0001, and scale bar = 100 µm.

To investigate the therapeutic potential of quercetin in orthodontic settings, we established a mouse model of OTM. Following the application of standardized orthodontic force, animals were treated with either quercetin at varying doses or a vehicle control. Histopathological evaluation via hematoxylin and eosin (H&E) staining revealed that quercetin treatment significantly attenuated inflammatory cell infiltration and preserved the structural integrity of the periodontal ligament compared to the control group (Figure 1A). Concomitantly, quercetin administration led to a marked, dose‐dependent reduction in the local expression of key pro‐inflammatory cytokines, IL‐6 and TNF‐α (Figure 1B). Consistent with its anti‐inflammatory effect, TRAP staining demonstrated a significant decrease in osteoclast number and activity at the alveolar bone surface in quercetin‐treated mice (Figure 1C). Micro‐CT analysis corroborated these findings, showing that quercetin effectively inhibited orthodontic force‐induced ABL and preserved bone volume (Figure 1D).

### 3.2 Quercetin Suppresses the IL‐17‐Mediated Inflammatory Cascade and Osteoclastogenesis

**Figure 2:**
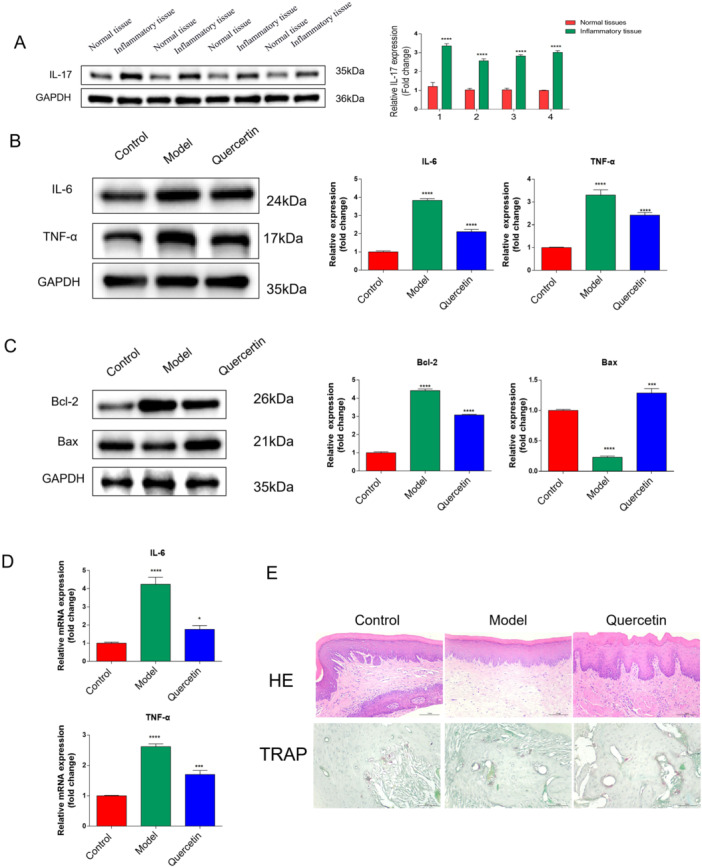
Quercetin can effectively inhibit the progression of the OTM model. (A) IL‐17 is overexpressed in OP models. (B) The expression levels of inflammatory factors in the quercetin treatment group were significantly reduced. (C, D) The expression of inflammation‐specific genes was measured by real‐time PCR in preosteoclastic RAW264.7 cells after quercetin and LPS administration. (E) H&E and TRAP staining of tissue samples of the animal model before and after treatment. Data are mean ± s.e.m., *n* = 3, *p < 0.05, ***p < 0.001, ****p < 0.0001, and scale bar = 100 µm.

Given the pivotal role of IL‐17 in inflammatory bone destruction, we assessed its involvement. In our OTM model, IL‐17 expression was significantly upregulated. Quercetin intervention potently downregulated both systemic and local levels of IL‐17 and its downstream inflammatory mediators (Figure 2A). In vitro, using the LPS‐stimulated RAW264.7 preosteoclastic cell model, quercetin significantly inhibited the expression of inflammation‐specific genes (Figure 2B) and effectively suppressed RANKL‐induced osteoclast differentiation, as evidenced by TRAP staining (Figure 2C). This aligns with its known capacity to modulate NF‐κB and MAPK signaling pathways.

### 3.3 Quercetin Exerts Its Protective Effects by Inhibiting the Hippo/YAP Signaling Pathway

**Figure 3:**
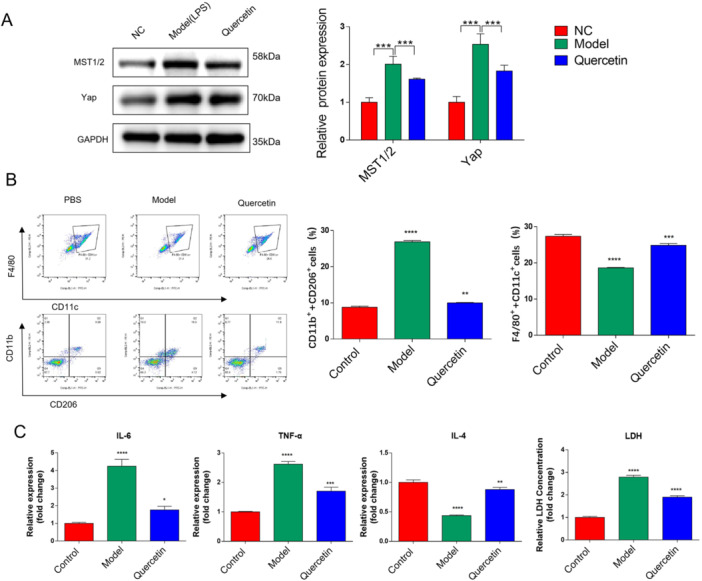
Hippo/YAP signaling pathway is abnormally activated in the periodontal tissues of orthodontic‐induced periodontitis. (A) The expression of Hippo/YAP in OP models. (B) Quercetin significantly inhibited the apoptotic level of cells induced by LPS. (C) ELISA test results for the expression of inflammatory factors in each group of cells. Data are mean ± s.e.m., *n* = 3, *p < 0.05, **p < 0.01, ***p < 0.001, and ****p < 0.0001.

Mechanistic exploration focused on the Hippo/YAP pathway, a key regulator of inflammation and bone homeostasis. We found that quercetin normalized the pathological overactivation of Hippo/YAP signaling induced by LPS in vitro (Figure 3A). At the molecular level, RT‐qPCR and Western blot analyses revealed that quercetin dose‐dependently inhibited YAP mRNA expression and nuclear translocation, while promoting the cytoplasmic retention of phosphorylated (inactivated) YAP in RAW264.7 cells (Figure 3B). This inhibition of YAP activity disrupted downstream pro‐apoptotic and pro‐inflammatory signaling cascades (Figure 3C).

### 3.4 Quercetin Modulates Cellular Functions and the Bone Microenvironment via YAP

The functional consequences of YAP inhibition were assessed through a series of cellular assays. Quercetin treatment significantly inhibited the proliferation of preosteoclasts, reducing colony formation capacity (Figure 4A, *p* < 0.001). Flow cytometry analysis further showed that quercetin induced cell cycle arrest at the G0/G1 phase and promoted apoptosis (Figure 4B,C). In the bone metabolic context, quercetin exhibited a dual regulatory function: it enhanced osteogenic differentiation, as indicated by increased ALP activity and OCN secretion (*p* < 0.01), while simultaneously inhibiting osteoclastogenic activity (Figure 4D). Furthermore, quercetin treatment significantly reduced the intracellular levels of reactive oxygen species (ROS) (Figure 4E, *p* < 0.001) and decreased the pro‐inflammatory ratio of RANKL/OPG in the cellular microenvironment (Figure 4F). Critically, the co‐administration of Verteporfin, a specific YAP inhibitor, completely abolished all the aforementioned biological effects of quercetin, confirming that its actions are specifically mediated through the inhibition of the Hippo/YAP signaling axis (Figure 4G).

Figure 4Quercetin inhibits the progression of the OTM model by acting through the YAP signaling pathway. (A, B) RT‐qPCR and WB were used to detect the inhibitory efficiency of YAP siRNA. (C) The clone formation assay was used to detect cell proliferation. (D, E) Flow cytometry is used to detect cell apoptosis and the cell cycle. (F) The enzyme‐linked immunosorbent assay was used to detect the levels of inflammatory cytokines and LDH release in the cells. (G) Alizarin Red S was used to detect the Osteogenic differentiation level. The DCFH‐DA reactive oxygen species (ROS) fluorescent probe is used to detect the content of ROS in cells. Detection of osteoclast differentiation in rat macrophage cell lines using TRAP. (H) Micro‐CT detection of ABL levels in the rat mandible. Data are mean ± s.e.m., *n* = 3, ***p < 0.001, ****p < 0.0001, compared with the LPS group, ^#^ *p* < 0.05, ^##^ *p* < 0.01, ^####^ *p* < 0.0001, and scale bar = 100 µm.

**Figure:**
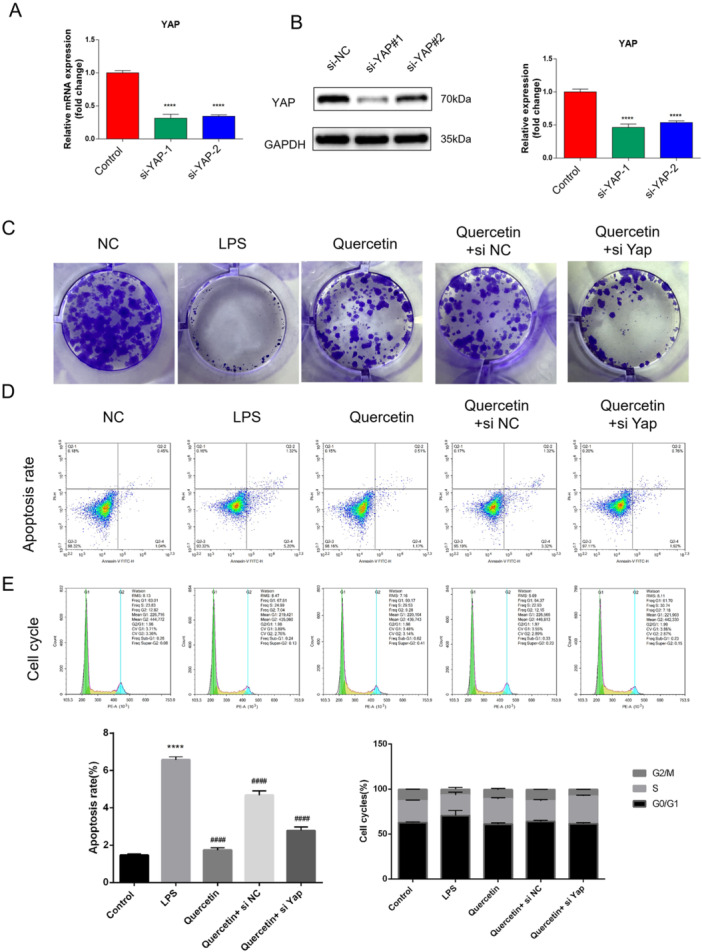

**Figure:**
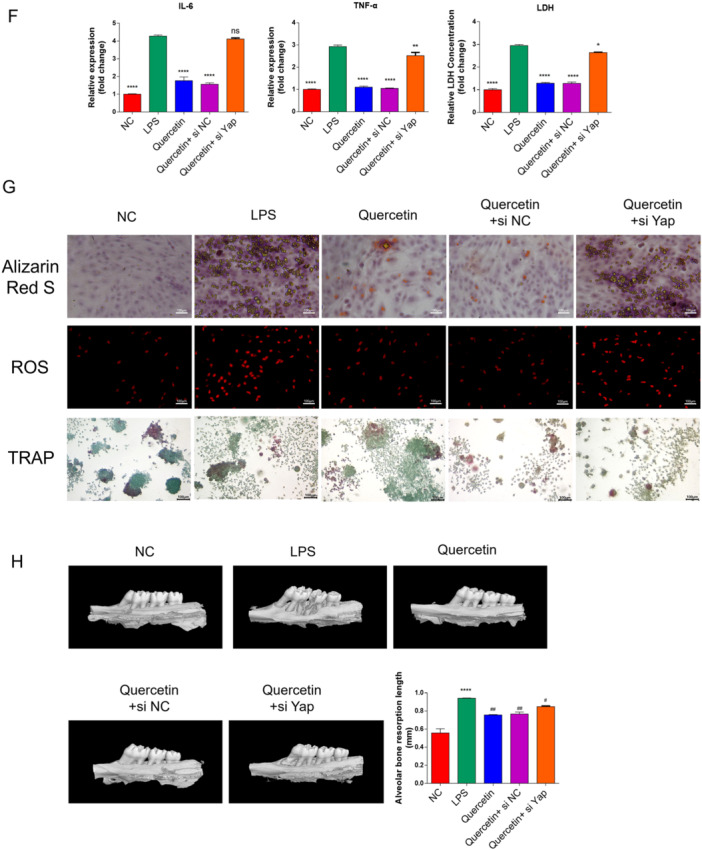

## 4 Discussion

This study elucidates a novel mechanism by which quercetin mitigates OTM‐associated periodontitis: suppression of macrophage aerobic glycolysis via the IL‐17/Hippo/YAP signaling axis. Our findings establish a direct link between immune signaling and metabolic reprogramming in periodontal inflammation, positioning quercetin as a dual‐acting therapeutic agent that targets immune‐metabolic crosstalk. The present study identifies a novel mechanism by which quercetin, a natural flavonoid, alleviates OTM‐associated periodontitis: targeting the IL‐17/Hippo/YAP signaling axis to suppress macrophage aerobic glycolysis. This finding expands understanding of immunometabolic crosstalk in periodontal inflammation and highlights quercetin's potential as a therapeutic adjunct for improving orthodontic outcomes.

OTM‐associated periodontitis is driven by mechanical stress‐induced immune dysregulation, with pro‐inflammatory (M1) macrophages relying on aerobic glycolysis to sustain inflammation. While quercetin's anti‐inflammatory effects in chronic inflammation are established, our study is the first to link it to the IL‐17/Hippo/YAP axis—an emerging regulator of immunometabolism. Mechanical stress during OTM upregulates IL‐17 in periodontal tissues, which activates the Hippo/YAP pathway in macrophages; specifically, IL‐17 induces YAP nuclear translocation, driving aerobic glycolysis via upregulation of glycolytic enzymes (HK2, LDHA, and PKM2).

A key contribution is identifying the Hippo/YAP pathway as a critical mediator of IL‐17‐driven macrophage glycolysis. In vitro experiments in THP‐1 macrophages confirm that IL‐17 promotes YAP nuclear localization and glycolytic enzyme expression, effects reversed by quercetin. YAP KO macrophages show blunted glycolytic responses to IL‐17, and quercetin fails to further inhibit glycolysis in these cells—confirming YAP's indispensability in this pathway. This contrasts with YAP's context‐dependent roles in other settings, underscoring quercetin's ability to fine‐tune macrophage metabolism without global Hippo pathway suppression.

Quercetin's suppression of macrophage glycolysis is accompanied by a metabolic shift toward oxidative phosphorylation (OXPHOS), associated with anti‐inflammatory (M2) polarization (Zhang et al. 2024; An et al. 2024; Li et al. 2024). This reprogramming correlates with reduced pro‐inflammatory cytokines (IL‐6, TNF‐α) and increased reparative markers (Arg‐1, IL‐10), restoring macrophage homeostasis in the inflamed periodontium (Zeng et al. 2024). Notably, quercetin acts in a concentration‐dependent manner, with lower doses (0.1–1 μM) targeting glycolysis and higher doses (10 μM) inducing broader metabolic suppression—emphasizing the need for dose optimization, potentially via topical delivery to minimize systemic toxicity.

Translationally, quercetin addresses a critical orthodontic challenge: mitigating inflammation without impeding bone remodeling (a limitation of current NSAID therapies). Its dual action—suppressing pathologic inflammation while preserving bone homeostasis—makes it ideal for local application (e.g., in adhesives or mouth rinses) during OTM. Additionally, YAP levels in gingival crevicular fluid may serve as a biomarker for treatment monitoring, though species‐specific differences and clinical validation are needed.

Limitations include the study's focus on acute OTM‐induced inflammation (vs. chronic polymicrobial periodontitis), its emphasis on macrophages (with other immune cells potentially contributing), and the need for long‐term safety data on oral quercetin delivery. Future research should address these gaps to advance clinical translation.

In summary, this study establishes quercetin as a multifaceted inhibitor of OTM‐associated periodontitis, acting through the IL‐17/Hippo/YAP axis to suppress macrophage aerobic glycolysis. By bridging immune signaling, metabolic reprogramming, and bone biology, our findings advance metabolic immunomodulation as a therapeutic strategy in orthodontics, paving the way for quercetin‐based interventions to enhance treatment efficacy and safety.

## Author Contributions

F.Y., Z.S., and Z.L.: Responsible for the study designing, research fields, drafting, and finalizing the paper; B.L., R.X., and Y.Z.: Wrote the manuscript and drew the pictures, collected, and organized literature; Y.Z. and Z.L.: Proofread the manuscript.

## Ethics Statement

All procedures performed in the studies involving animals were in accordance with the ethical standards of the Institutional Animal Care and Use Committee (IACUC) of The Affiliated Stomatological Hospital of Jiangxi Medical College.

## Conflicts of Interest

The authors declare no conflicts of interest.

## Data Availability

All processed data and models used during the study are available from the corresponding author upon request.
